# VENturing into machine learning for the morphological analysis of von Economo neurons

**DOI:** 10.1038/s41598-025-30470-y

**Published:** 2025-12-20

**Authors:** Ivan Banovac, Oliver J. Bruton, Luis Mercado-Díaz, Julian Tejada, Fernando Marmolejo-Ramos

**Affiliations:** 1 Department of Anatomy and Clinical Anatomy, and Croatian Institute for Brain Research, University of Zagreb School of Medicine, Zagreb, Croatia; 2 Department of Psychology, School of Medicine and Health Sciences, Carl von Ossietzky Universität Oldenburg, Oldenburg, Germany; 3 Department of Biomedical Engineering, College of Engineering, University of Connecticut, Storrs, CT USA; 4 Department of Psychology, Federal University of Sergipe, São Cristóvão, Brazil; 5 College of Education, Psychology, and Social Work, Flinders University, Adelaide, SA Australia

**Keywords:** Cerebral cortex, Specialized neurons, Machine learning, Neural network, Neuronal morphology, Cellular neuroscience, Computational neuroscience

## Abstract

Von Economo neurons (VENs) are a specialized type of large, highly elongated projection neurons located in specific cortical regions. Despite their implication in higher-order cognitive functions and psychiatric disorders in humans, consistent and objective identification criteria for VENs remain lacking. We analyzed 761 digitally reconstructed neurons from the NeuroMorpho.Org database. We applied six supervised machine learning algorithms and a convolutional neural network with Grad-CAM visualization to classify the reconstructions into VENs and pyramidal neurons. Variable importance was evaluated using information-driven and expert-based selection. We compared the classifications made by machine learning algorithms to the reconstructions’ original labels. Reconstructions misclassified by the classifier models were further examined by a neuroanatomy expert. Machine learning models generally achieved high classification accuracy. Morphometric features such as dendritic length and number of stems emerged as some of the key discriminators. Expert ratings only partially aligned with machine findings, and there was low agreement between experts. Most misclassifications made by the classifier models were attributable to reconstruction artifacts or ambiguous morphology rather than model limitations. Our findings demonstrate the utility of combining machine learning with expert insight for distinguishing VENs from pyramidal neurons. While soma shape remains important for the characterization of VENs, classifier models revealed that dendritic architecture may be equally as specific and could help distinguish between borderline cases. This framework offers a replicable, data-driven method for studying VENs and can be utilized for future research on their distribution and function.

**Supplementary Information:**

The online version contains supplementary material available at 10.1038/s41598-025-30470-y.

## Introduction

In the course of the last decades the study of the human brain has experienced tremendous progress. Owing to the advent and continuous development of various magnetic resonance imaging techniques (e.g. fMRI), it has become possible to describe complex behavioral traits in terms of putatively underlying macro-neurological variables in vivo (such as large-scale functional networks;^1,2^). However, a comprehensive account of the latter crucially hinges on a thorough grasp of their microstructural constituents^3–5^. The brain is estimated to contain up to 86 billion neurons^6^. Beyond their sheer number however, they are also distinguished by an array of features (e.g., molecular, morphological, electrophysical, functional;^4,7^). Moreover, they evince considerable variability regarding these facets—not only across cortical regions, but also across subjects^8,9^. Consequently, this complexity has prompted attempts to classify neurons in terms of a discrete number of subtypes or classes^7^.

Although the basic idea behind this approach dates back to the early work of pioneers such as Ramon y Cajal^10^, a solid, let alone exhaustive understanding of how neuron types can be reliably identified and how they may contribute to higher-order behavior, remains lacking^7,11,12^. This comes as no surprise, given that the study of neuronal subtyping faces multiple challenges. These include non-overlapping classification schemes and considerable discrepancies pertaining to basic terminology and methodological approaches^12,13^. Regarding the aspect of morphology, one of the prime examples of how such factors may affect the classification process, has manifested itself in the case of a neuron first detailed by Austrian-Romanian psychiatrist and neurologist Constantin von Economo^14,15^. His analysis of the human brain’s cytoarchitecture led to the description of a nerve cell with salient morphological features, including a large, extremely elongated (stick-like) cell body^15,16^. Furthermore, the presence of these “corkscrew cells” appeared to be primarily restricted to layer Vb of the anterior cingulate cortex (ACC) and the fronto-insular cortex (FI) where they were frequently observed in assemblages of 3 to 6 neurons^17,18^. Consequently, they were later recognized as a separate class of neurons, which, to honor their discoverers memory, have been dubbed “von Economo neurons” (VENs;^19^).

Subsequent studies reported the identification of VENs in brain areas other than the ACC and FI such as the dorsolateral prefrontal cortex (DLPFC), the frontal pole, as well as the posteromedial cortex/precuneus^20–22^. Moreover, they have been implicated in the origin of psychopathologies including schizophrenia, frontotemporal dementia (FTD) and autism^23–25^. Such findings have given rise to speculative accounts as to the functional significance of VENs, ranging from interoception to general intelligence^1,26^. However, as pointed out by Petanjek et al.^27^, several studies on VENs are based on the analysis of more frequently occurring spindle-shaped bipolar neurons. Since the morphological features of the latter deviate from the initial corkscrew cells’ appearance, their identification as VENs has been called into question. Nevertheless, they are often subsumed under the same umbrella term, sparking a recent debate as to what “makes a VEN a VEN”.

The accurate classification of VENs is not merely a matter of taxonomic precision. Misclassification of morphologically similar neurons as VENs is likely to lead to inaccurate conclusions regarding their prevalence, distribution, and selective vulnerability. This can confound studies investigating the evolutionary origins of VENs and makes determining the functional roles of these cells more challenging. In addition, misclassification of VENs severely limits their use as potentially valuable histopathological markers. For example, if studies implicating VENs in neuropathological conditions have used inconsistent or poorly defined criteria to link selective VEN loss to a given pathology, the validity of such findings becomes questionable and their reproducibility severely compromised. Therefore, it is essential to establish robust, reproducible criteria for VEN identification to ensure the validity, reproducibility, and comparability of future research in this domain.

Currently, there is still an absence of a standardized classificatory process, not only for VENs, but even for neuronal morphology more broadly^27^. We contend that a way to alleviate this problem, in part, lies in devising a more objective technique of differentiating VENs from similar spindle cells, which fall within the category of “common modified pyramidal neurons” (MPN;^13,28^). To this end, we employ various machine learning algorithms, training them on a batch of VENs as well as VEN-like cells obtained from samples of post-mortem brain tissue available in the NeuroMorpho.Org database^29^. The aim of this study is to generate a data-driven classificatory procedure of high accuracy, that may assist future researchers in the decision whether or not to classify any given neuron as a VEN. We expect this to greatly enhance the understanding of the VENs’ precise cortical distribution patterns and ultimately, their functional significance.

## Methods

### Neuronal reconstructions from NeuroMorpho.Org

**Fig. 1:**
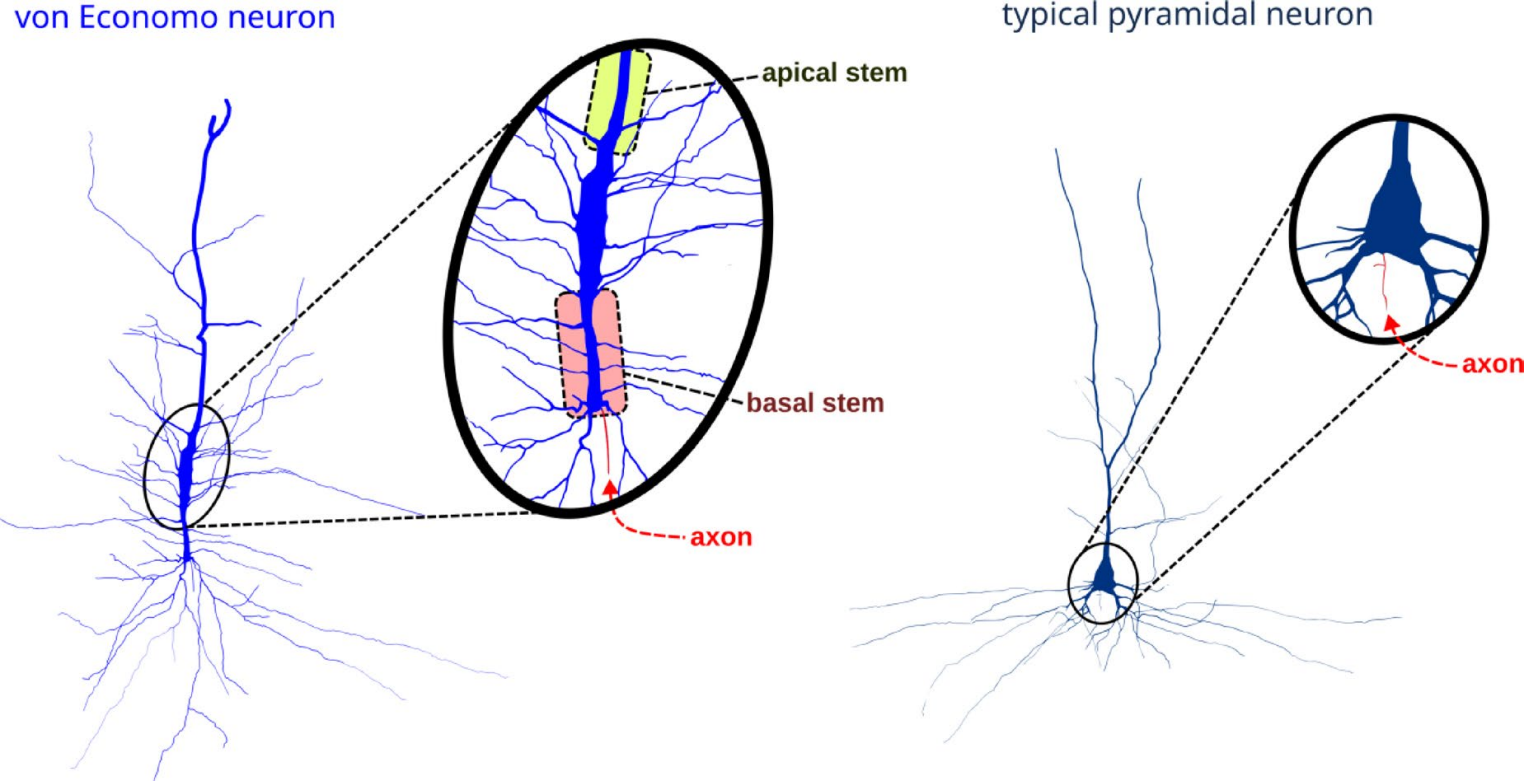
Comparison of the morphology of a typical von Economo neuron (VEN) and a typical pyramidal neuron (image modified from^28^, Fig. S3). The following key morphological features of VENs are visible: (1) an elongated, stick-shaped cell body, (2) lack of clear demarcation between cell body and main apical and basal processes (apical and basal stems), (3) axon originating from distal end of the basal stem, (4) basal stem terminally branching into multiple significantly thinner branches in a brush-like pattern (called a basal tuft/skirt). Unlike the VEN, a typical pyramidal neuron has a pyramidal cell body, clear demarcation between the cell body and the main dendrites, and an axon arising from the basal side of the cell body, rather than distally from the basal processes.

A total of 761 digital neuronal reconstructions^19,28,30,31^, 706 classified as pyramidal neurons and 55 classified as VENs (see Fig. 1 for morphological comparison of typical VENs and pyramidal neurons), were retrieved from NeuroMorpho.Org repository (RRID:SCR_002145) in March 2024. NeuroMorpho.Org is a publicly accessible repository of previously published morphological datasets. No new experiments, tissue collection, or human-subject procedures were performed in this study. All reconstructions were originally generated, anonymized, and deposited by the contributing laboratories together with the appropriate ethical approvals and consent procedures, as required by the original studies and their institutional ethics committees. In the present work, we conducted only secondary analyses of these openly available datasets. The analyzed dataset constituted at that time all available morphological reconstructions of human pyramidal cells under the keywords: *insula*, *fronto-insula* and *anterior cingulate*. All reconstructions were retrieved in SWC format, a text-based file describing the three-dimensional morphology of neurons or glia as a vectorized tree structure consisting of a series of connected nodes. Each node is represented by a vector of seven values. The first value is an ID number, generally starting from 1 up to the maximum number of nodes. The second is an integer number ranging from 0 to 10 that represents the structure identifier, for example, number 1 identifies a section that is part of the soma, number 2 a section that is part of the axon, and so on. The third, fourth and fifth values represent the ‘x’, ‘y’ and ‘z’ spatial coordinates in micrometers, respectively. The sixth value represents the radius of the section (e.g. dendritic thickness). Finally, the seventh value represents the parent ID number of the section to which the current section is connected^32^. Each section is represented as a cylinder compartment that starts at a particular set of Cartesian coordinates (x, y, and z) and has a corresponding diameter. The compartment extends until it reaches the next set of coordinates, where the diameter is usually smaller.

### Morphological parameters analyzed in the study

A set of 21 morphometric parameters were estimated from each SWC file model using L-measure^33^, see Supplementary Table 1. The description of these metrics is available on the L-measure website (http://cng.gmu.edu:8080/Lm/) and presented in Supplementary Table 1. In addition to the 21 measurements provided by the L-measure software, the height-to-width ratio of the cell body was calculated and used as an additional parameter. Mann–Whitney’s test was used to compare the measurement values between correctly and incorrectly classified reconstructions. Bonferroni correction was done to control for family-wise error rate that occurs when performing multiple hypotheses tests.

### Variable selection approach

Once the measurements were estimated for each morphological reconstruction, the next step was a variable selection procedure based on variable importance. Variable importance evaluates how much a model changes when a variable or a group of variables are removed (see^34,35^). For this, we built a model considering the cell class (pyramidal or VEN) as outcome and the 21 measurements as predictors, and tested with six supervised machine learning classification algorithms: BART, C5.0, random forest, XGB, SVM, and EARTH, available on the R package MachineShop.

#### Supervised machine learning classification algorithms

Bayesian additive regression (BART)^36^ is a statistical model for classification that merges multiple “weak” *decision trees* using a Bayesian approach. This integration results in a flexible and powerful predictive model. BART employs a priori distribution to regularize the trees and prevent overfitting and utilizes Markov Chain Monte Carlo (MCMC) methods for estimation. A *decision tree* is a classification algorithm that works by recursively partitioning data based on the values of its features (attributes) to predict a target variable (class).

C5.0^37^ is a classification algorithm that builds decision trees and rule sets by recursively splitting data into smaller subsets, aiming to minimize the variety of classes within each subset.

Extreme gradient boosting (XGBoost)^38^ is a machine learning algorithm that belongs to the ensemble learning family, specifically the boosting type. These models combine multiple “weak” learners (models that perform slightly better than random chance, such as decision trees) to create a “strong” learner with significantly improved performance following a gradient descent as optimization algorithm.

Random forest^39^ enhances decision trees by using multiple randomly sampled subsets of the training data to create an ensemble of trees—a technique called Bagging (bootstrap aggregating)^40^. Further enhancing randomness, each tree considers only a random subset of features at each node. The final prediction is determined by aggregating the predictions of call trees, using majority voting for classification.

Support vector machine (SVM)^41^ is another classification algorithm that seeks to identify the optimal hyperplane for separating data points into different classes. SVM maximizes the margin, which is the distance between the hyperplane and the nearest data points (support vectors), thereby improving generalization and minimizing classification errors.

EARTH is an R and Python implementation of Multivariate Adaptive Regression Splines (MARS)^42^, a non-parametric regression technique. MARS uses piecewise linear functions, called basis functions, to predict the outcome variable. These functions are selected through a two-stage process: a forward pass, which adds functions that minimize the residual sum of squares (RSS), and a backward pass, which removes less effective functions based on their contribution to the generalized cross-validation (GCV) score. The final model is a linear combination of the selected basis functions.

#### Other classification methods

In addition to the supervised machine learning classification algorithms, which were trained using morphological measures, we also used a Gradient-weighted Class Activation Mapping (Grad-CAM) convolutional neural network technique—an algorithm that can use images as input to identify which parts of the image are most important for a convolutional neural network’s (CNN) prediction^43^. Grad-CAM identifies the important regions in an image by looking at how much the output for a specific class changes when we slightly change the activations of the feature maps in the last convolutional layer (see Supplementary material). The gradients give information on which feature maps are most sensitive to changes that affect the target class. By weighing the feature maps with these gradients, we created a heatmap that highlighted the relevant regions.

### Supervised machine ensemble learning classification algorithms and expert opinion survey

The integration of machine learning with prior or expert knowledge has emerged as a promising approach to address the limitations of purely data-driven models. As highlighted by von Rueden et al.^44^, machine learning can have limitations when dealing with insufficient training data, and incorporating prior knowledge can lead to more robust and trustworthy models. This approach, termed “informed machine learning,” explicitly integrates formal representations of knowledge into the learning process, allowing models to generalize better with less data. Similarly, Gennatas et al.^45^ introduced “expert-augmented machine learning,” which automatically extracts and incorporates (clinical) expert knowledge into machine models, demonstrating improved performance on out-of-sample data with less training data. Steyvers et al.^46^ further explored human-AI complementarity through a Bayesian modeling framework, showing that hybrid combinations of human and machine classifiers can outperform either working alone, even when they perform at different accuracy levels. These approaches highlight that the optimal learning strategy often involves combining the complementary strengths of humans and machines rather than relying solely on data-driven methods or expert systems^44–46^. By incorporating expert knowledge, machine learning models become more data-efficient, robust to distribution shifts, and aligned with human understanding. In this study, we used a combination of machine learning results and human expert knowledge to classify the relevant morphological features of VENs.

Due to the computational cost of the supervised machine learning classification algorithms it was not possible to run the model including all 21 measurements at the same time. The MachineShop algorithms implementation in R only worked well with a maximum of ten variables. For this reason, we adopted a bootstrapping approach, selecting 10 variables randomly at a time, and repeating the procedure 5000 times.

At the end, the variables that appeared most often in the first position of importance were considered to form the final classification model.

The results of the information-driven variable selection (performed by the *Supervised machine ensemble learning classification algorithms*) were compared with a variable selection made by a group of nine experts through an online form provided for this purpose. The survey was programmed using a set of java-script open-source libraries (https://github.com/surveyjs) and made available using JATOS^47^ on MindProbe server (https://mindprobe.eu/). The survey consisted of five demographic questions (1. *Do you have experience with digitally reconstructed neurons?* 2. *If so*, *what software do you use to digitally reconstruct neurons?* 3. *Have you ever heard of von Economo neurons?* 4. *Do you know the web-accessible archive of digital reconstruction of neural morphology NeuroMorpho.Org?* 5. *From 0 (no expertise) to 10 (high expertise), how much expertise do you have in neuromorphology?*) and then the 21 morphological features available on NeuroMorpho.Org were listed (in a random order for each participant), and the last question consisted of ranking each feature from most to least important in characterizing a pyramidal neuron.

Before responding, participants read the participant information sheet and agreed to participate. Their participation was anonymous, and the survey was approved by the Human Research Ethics Committee of the University of South Australia (approval no. 205999) and conducted according to the principles expressed in the Helsinki Declaration.

#### Classification of pyramidal neurons and VENs

After selecting the variables, a set of seven different classifiers (BART, C5.0, Random Forest, XGB, SVM, EARTH and Grad-CAM) were used to evaluate which VEN morphologies are the most difficult to classify. To facilitate comparison, due to the discrepancy between the number of pyramidal and von Economo neurons available in NeuroMorpho.Org, the models were trained with subsamples of 50 cells from each group and the process was repeated 5000 times to ensure that all features of all models were evaluated by the machine learning models. Lastly, the morphological characteristics of the reconstructions that had been most misclassified by all models were studied.

##### Convolutional neural network classification

For the classification of reconstructions into pyramidal neurons or VENs, we implemented a transfer learning approach utilizing the VGG16 architecture with batch normalization (VGG16-BN)^48^. VGG16 was selected due to its proven capability in feature extraction and its relatively simple architecture that facilitates interpretation of results. The network consists of thirteen convolutional layers organized in five blocks, followed by three fully connected layers. Each convolutional layer employs 3 × 3 kernels with stride 1 and padding 1, maintaining spatial resolution throughout the feature extraction process^48^.

We modified the original VGG16-BN architecture by replacing the final fully connected layer, originally designed for 1000-class ImageNet classification, with a binary classification layer suitable for our VEN vs. pyramidal neuron discrimination task. This adaptation preserved the rich feature hierarchy learned from ImageNet, while allowing specialization for our specific classification problem^49^. The network was initialized with pre-trained weights from ImageNet, employing transfer learning to leverage general visual features learned from a large-scale dataset^50^.

Each neuronal reconstruction was rendered as a two-dimensional projection in three different ways: (1) original screenshot of the neuronal morphology from NeuroMorpho.Org; (2) screenshot of the SWC file using the HBP Neuron Morphology online viewer^51^; (3) and the screenshot of the SWC file using the NEURON software 3D import tool^52^ with the “show diameter” option checked. In all cases, the screenshots represent the default visualization of the neuron morphology without any rotation. Two additional visualizations were used because these platforms display certain morphological features differently than the NeuroMorpho.Org screenshots. Specifically, the HBP Neuron Morphology Online Viewer highlights the soma, which is typically rendered larger in this viewer compared to the NeuroMorpho.Org representation, and for this reason these representations were called *Soma-Focused*. The NEURON software 3D import tool permits the visualization of the dendrite diameter, which is why they were called *Diameter-Enhanced*. Finally, the NeuroMorpho.Org representations were called *Original*. Each neuron have a version masked that allowed to understand what regions of SOMA or Dendrites where more relevant in the classification.

The images were preprocessed using standard computer vision techniques and resized to 224 × 224 pixels to match VGG16’s input requirements. To maintain consistency with the pre-trained network’s expectations, images were normalized using ImageNet statistics (mean = [0.485, 0.456, 0.406], std = [0.229, 0.224, 0.225])^53^.

##### Training protocol and optimization

The network was trained using the Adam optimizer^54^ with an initial learning rate of 1e-4 and default momentum parameters (β1 = 0.9, β2 = 0.999). Learning rate decay was implemented using a cosine annealing schedule^55^. The loss function employed was binary cross-entropy:

L = -[y log(p) + (1-y)log(1-p)].

where *y* represents the true label and *p* the predicted probability. Training was conducted for 10 epochs with a batch size of 16, optimized for available computational resources while maintaining training stability.

## Results

### Information-driven versus human-driven variable selection

**Fig. 2:**
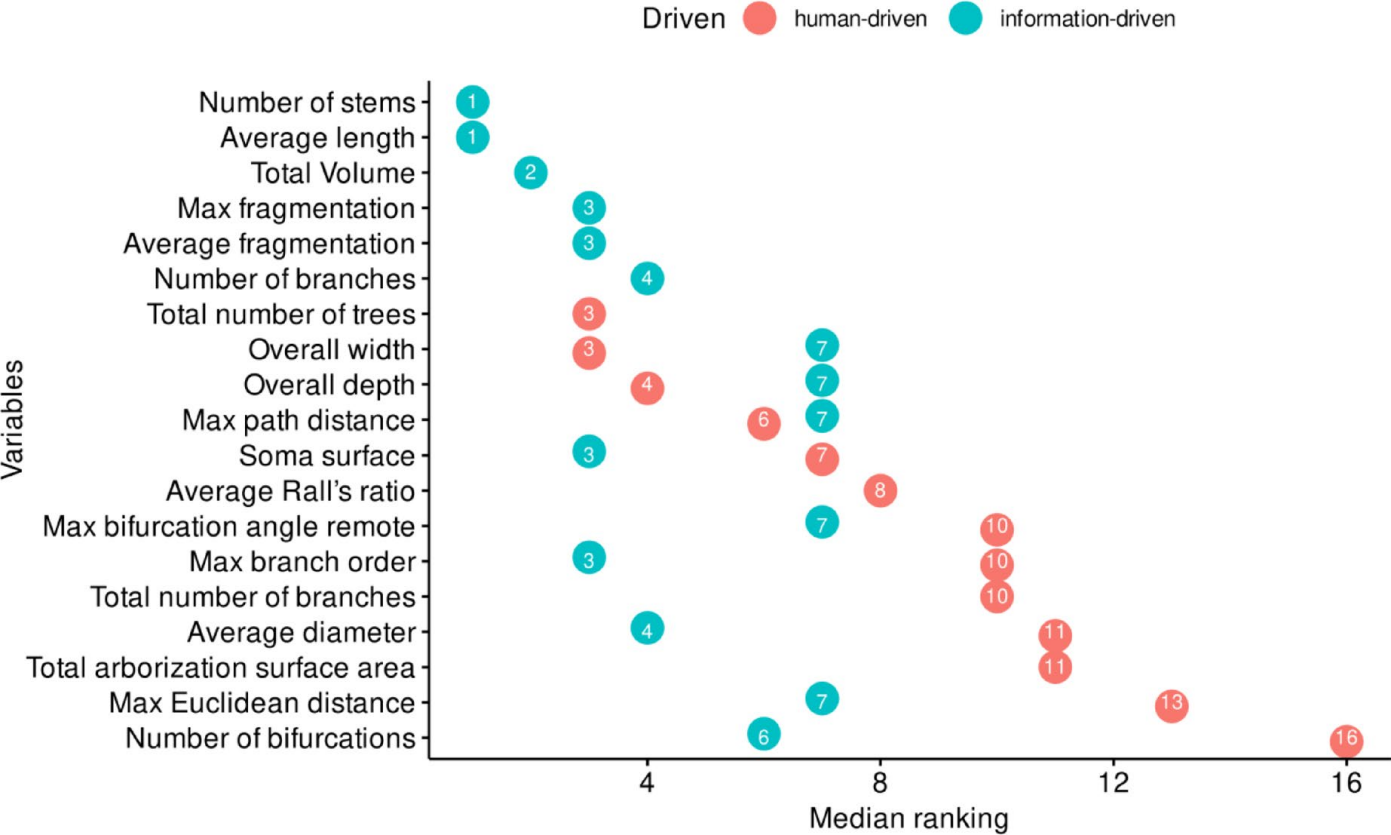
Most important variables found in human and information driven variable selection. The median ranking is represented by the *x* values and the numbers assigned to each point. A lower value indicates a higher importance of the variable. For example, the most important variables for all machine learning classifiers were *average length* and *number of stems*, but this level of agreement was not observed between the human experts, for whom the variable with the best average ranking was *total number of trees*, which appeared in seventh place for the machine learning classifiers. The interrater agreement between experts’ response was low (Fleiss’ Kappa for *m* raters; number of variables = 19, experts = 7, Kappa = 0.0132, *z* = 1.12, p = 0.262). Spearman rank correlation between human-driven and information-driven rankings: *ρ* = 0.28, *p* = 0.24 (indicating low agreement between methods).

**Fig. 3:**
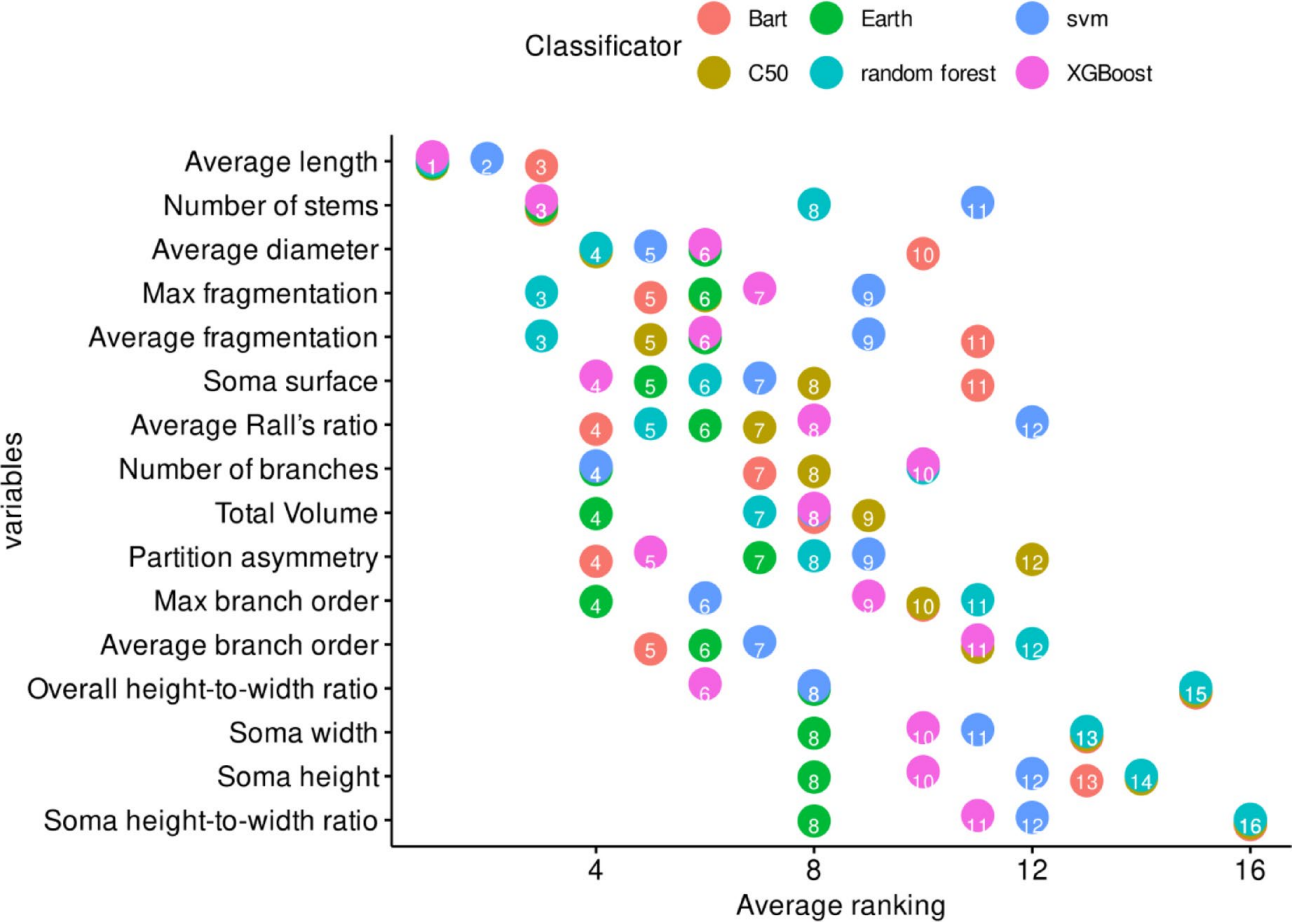
Most important variables considering the classification algorithm. The importance of variables is represented by their median ranked order (*x* values and point numbers), where lower values indicate higher importance. Across all machine learning classifiers, average length emerged as the most significant variable. Specifically, it held the top spot in importance for C50, Earth, and XGBoost, and was the second most important variable for SVM. Inter-algorithm correlations for variable importance rankings ranged from r = 0.78 to r = 0.94 (all *p* < 0.001), demonstrating high consistency among machine learning methods. Average inter-algorithm correlation: r = 0.87 ± 0.05.

The top ten variables found after information-driven variable selection were: *Average length, Overall width, Number of stems, Total number of trees, Average diameter, Overall depth, Average fragmentation, Max path distance, Max fragmentation, Overall height, Average Rall’s ratio, Average Contraction, Soma surface, Soma surface, Number of branches, Average Rall’s ratio, Partition asymmetry, Total number of branches, Total Volume, Max branch order and Average branch order.* Table 1 shows a list of the variables and their ranking obtained by each method. It highlights that only three of the most important variables (in bold) were identified as important by both the human-driven and information-driven variable selection methods. Figure 2 presents a comparison of the average ranking obtained by human-driven and information-driven variable selection. Figure 3 compares the average ranking obtained for each machine learning classifier.

**Table 1:** Results of the variable importance ranking from the information-driven and human-driven variable selection.

Importance ranking	Information-driven variable selection	Human-driven variable selection
1	Average length	Overall width
2	Number of stems	Total number of trees
3	**Average diameter**	Overall depth
4	Average fragmentation	Max path distance
5	Max fragmentation	Overall height
6	**Average Rall’s ratio**	Average Contraction
7	**Soma surface**	**Soma surface**
8	Number of branches	**Average Rall’s ratio**
9	Partition asymmetry	Total number of branches
10	Total Volume	Max branch order
11	Average branch order	**Average diameter**

Variables identified as important by both human-driven and information-driven variable selection are shown in bold.

To quantify the relationship between human-driven and information-driven variable selection approaches, we conducted correlation analyses. The results revealed low agreement between expert raters (Fleiss’ Kappa = 0.0132) In contrast, inter-algorithm correlations among machine learning methods were substantially higher, ranging from r = 0.78 to r = 0.94 (all *p* < 0.001), with an average correlation of r = 0.87 ± 0.05, demonstrating high consistency among computational approaches. In variable importance selection, there was relatively low agreement between the expert-driven approach and the machine learning methods, likely due to the low agreement between expert raters (Spearman rank correlation *ρ* = 0.28, *p* = 0.24).

To further investigate the consistency differences between machine learning algorithms and human experts, we conducted a variance analysis of feature ranking positions. Supplementary Table 3 reveals that human experts exhibited substantially higher variance in feature importance rankings compared to machine learning algorithms across all ranking positions.

The variance analysis demonstrates that machine learning algorithms were 14.18 times more consistent than human experts in identifying important morphological features, with overall variance of 0.22 ± 0.07 for algorithms compared to 3.12 ± 0.23 for human experts. This finding supports the reliability of computational approaches for objective feature selection in neuronal classification tasks.

### Quality of supervised machine learning classifiers

The classification quality evaluated during the information-driven variable selection process revealed four reconstructions that were consistently misclassified by all seven algorithms: 03b_spindle4aACC, 24_VEN_rapid, 14_VEN_Cox and 27o_spindle19aFI. All four reconstructions were identified as VENs on the NeuroMorpho.Org website. Furthermore, 63 additional reconstructions (51 pyramidal and 12 von Economo) were misclassified by five of the six machine learning algorithms employed in the information-driven variable selection process. Supplementary Table 2 provides a summary of these results.

**Fig. 4:**
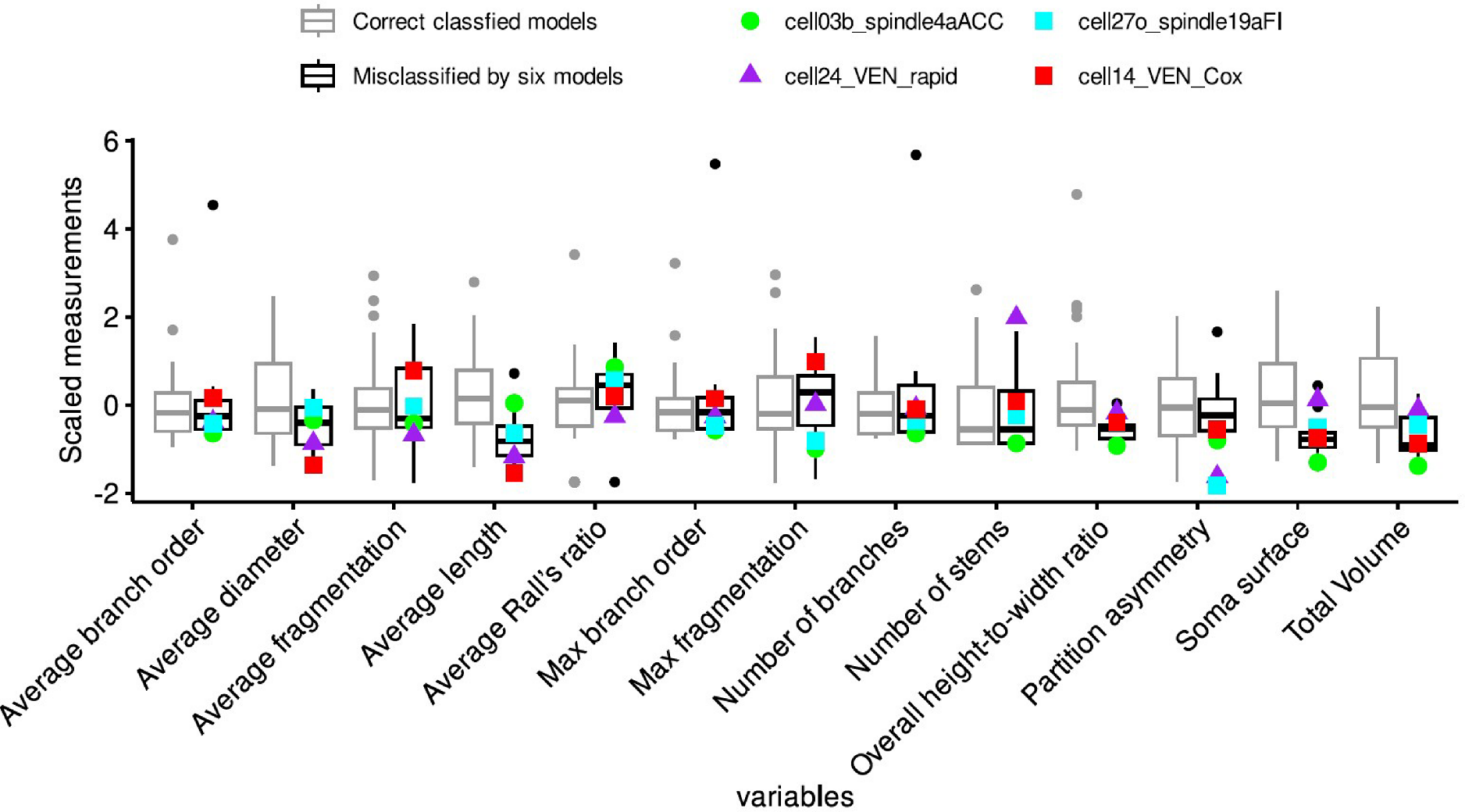
A comparison of the four misclassified reconstructions with the rest of VEN reconstructions from NeuroMorpho.Org, based on each of the neuromorphological measures used to characterize the reconstructions. To facilitate comparison, all measurements were scaled using the scale() command in R. The boxplots illustrate the distribution of these measures across all 55 VEN reconstructions, while the colored points highlight the specific measurement values for each of the four misclassified neurons, and the boxplot colors represent whether the cells were correctly or incorrectly classified. Each individual misclassified VEN reconstruction displayed certain values that substantially deviated from the average of correctly classified VEN reconstructions, however, Mann–Whitney U tests revealed no significant differences between misclassified and correctly classified VEN reconstructions as a whole.

Analysis of the neuromorphological measurements of the four reconstructions that were misclassified by all the algorithms revealed that these cells exhibit distinctive characteristics that differentiate them from other von Economo neurons available on NeuroMorpho.Org (see Fig. 4). When considering only the measurements that are projected outside the boxplots, the 24_VEN_rapid reconstruction presents a lower average length and partition asymmetry, as well as a higher number of stems. Conversely, the 03b_spindle4aACC model exhibits reduced soma surface area, total volume, and maximum fragmentation, while exhibiting elevated average Rall’s ratio. The 14_VEN_Cox reconstruction presents lower average length and diameter and higher average and maximum fragmentation. Finally, the 27o_spindle19aFI reconstruction exhibits a lower maximum fragmentation and average partition asymmetry. Thus, although each of these misclassified neurons displayed atypical values on certain measures, the pattern of deviation was not consistent across them.

**Fig. 5:**
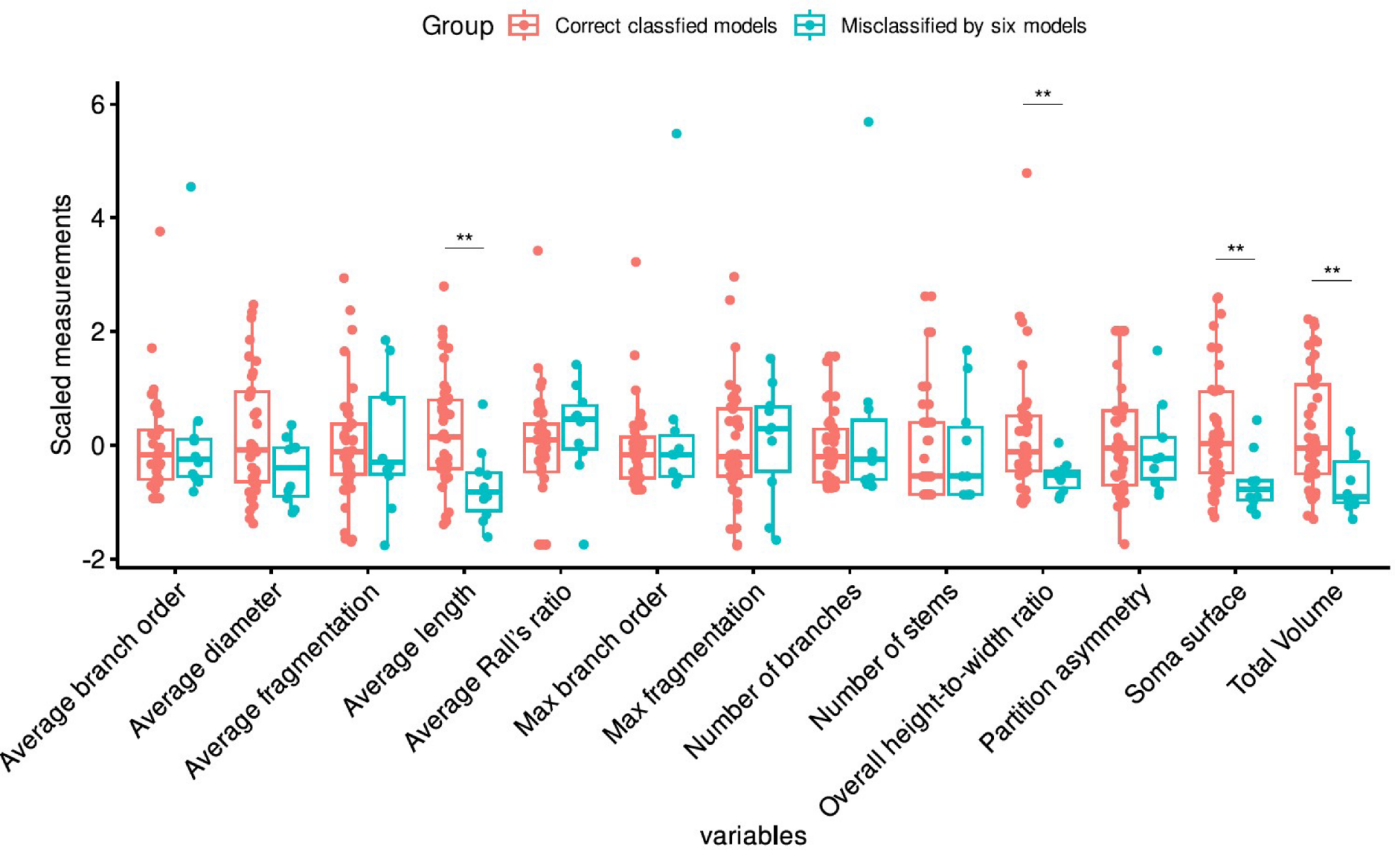
A comparison of the 11 misclassified reconstructions by at least six classifiers with the remaining reconstructions (52) on each of the neuromorphological measures used to characterize the reconstructions. To facilitate comparison, all measurements were scaled using the scale() command in R. Mann–Whitney U tests comparing the 11 misclassified reconstructions with the remaining reconstructions showed significant differences in: total volume (U = 327, *p* < 0.004), average length (U = 327, *p* < 0.003), soma surface (U = 322, *p* = 0.004), and overall height-to-width ratio (U = 325, *p* < 0.003). Effect sizes (Cohen’s d) ranged from 0.38 to 0.41.

Regarding the other 11 reconstructions misclassified by at least six classifiers, it is possible to state that most of them are smaller, in terms of length, diameter, volume and soma surface, compared to the remaining misclassified cells (Fig. 5).

### Most misclassifications occur due to methodological errors unrelated to the classifier models

Besides evaluating the morphometric characteristics of the misclassified cells, a detailed manual analysis of each of the reconstructions misclassified by 5 or more classifiers was performed by a neuroanatomy expert (IB). The analysis revealed that for most of the misclassified reconstructions we could find a reasonable explanation for such a high rate of misclassification that was unrelated to the classifier models. These explanations can be divided into the following categories: (1) errors in the reconstruction files, (2) methodological limitations, (3) ambiguous morphological features, and (4) errors in the original classification.

#### Errors in the reconstruction files

The first group of errors in the reconstruction files were errors that most likely arose due to problems with conversion between different file types. An example of this was the reconstruction 14_VEN_Cox where the soma was completely distorted to a globular shape in the .SWC file available in NeuroMorpho.Org (Supplementary Fig. 1A) but had a characteristic stick-shape in the original publication (see Fig. 2B in^28^). Besides the severe distortion of soma shape present in certain reconstructions, other reconstructions featured distorted transitions between the soma and first-order dendrites.

The second group of errors in the reconstruction files were errors that seem to be caused by human error during the reconstruction process—the most prominent one was lack of alignment in the Z-axis between the soma and one of the dendrites, probably caused by some kind of “Z drift” occurring during the reconstruction process. Reconstruction 24o_pyramidal17aFI is an example of this occurrence (Supplementary Fig. 1B).

#### Methodological limitations

For some reconstructions, it is possible that methodological limitations played a significant role in the classification process. It is well-known that the quality of Golgi impregnation can vary significantly^56^ and it seems that some reconstructions may have been derived from neurons with poor impregnation. A possible example for this would be the reconstruction 03b_spindle4aACC (Supplementary Fig. 1C)—this reconstruction can be difficult to evaluate properly because its dendritic arbor lacks complexity associated with either VENs or pyramidal neurons. It is possible that this could be due to incomplete impregnation of the dendritic arbor. Alternatively, this might be yet another cell subtype sufficiently different from regular VENs with complex dendritic arbors, so that the classifier does not identify it as a VEN.

In addition, some misclassified reconstructions appeared to have an apical dendrite that ended relatively near the soma. This is most likely due to the dendrite being cut on the edge of the histological section, which is a common methodological issue. It is also possible that the apical dendrite was deliberately not reconstructed in its entirety, since sometimes only the basilar dendritic tree is analyzed in the research. It is interesting to note that a short apical dendrite seemed to hinder correct classification for a lot of pyramidal neurons. Reconstruction D3_GIB3_13-10 is an example of a misclassified cell with a short apical dendrite (Supplementary Fig. 2A).

#### Ambiguous morphological features

Some reconstructions exhibited unusual or ambiguous morphological features, but it was not immediately clear why exactly they were misclassified. The most notable example was reconstruction 03b_pyramidal3aACC (Supplementary Fig. 2B), which was misclassified as a VEN, even though it seems to lack similarity to other cells classified as VENs. In this case, the unusual and ambiguous somatic morphology and dendritic topology might have contributed to the misclassification. Furthermore, such cells may be further evidence for the existence of a continuum in neuron morphology^57,58^.

#### Errors in the original classification

For a certain subset of reconstructions (11 in total, see Supplementary Table 2), the algorithm classified them as not-VENs, even though in the NeuroMorpho.Org database they were classified as VENs. However, upon inspection of these neurons by a human researcher, it became clear that at least some of these reconstructions may have been mislabeled in the database. The reconstruction 25o_spindle17aFI is the best example because it has an almost pyramidal soma, which is distinctly different from the stick-shaped soma of VENs (Supplementary Fig. 2C). In such cases, it seems the classifiers we used correctly identified the cell type, rather than this being a case of misclassification.

### Quality of* convolutional neural network classification*

The implementation of our VGG-based classification model revealed distinctive performance patterns across various neuronal representations. Most notably, the diameter-enhanced representations achieved superior classification metrics, with a test accuracy of 98.18%, precision of 0.96, and perfect recall (1.00), yielding an F1-Score of 0.98 and ROC-AUC of 0.98. The original neuronal representations, while demonstrating robust performance, showed slightly lower metrics with a test accuracy of 93.64%, precision of 0.89, and perfect recall, resulting in an F1-Score of 0.94. Soma-focused representations maintained comparable efficacy, achieving a test accuracy of 94.55%, precision of 0.92, and near-perfect recall of 0.98, with an F1-Score of 0.95.

Cross-validation analysis demonstrated remarkable stability across both twofold and fivefold protocols. In the twofold cross-validation, diameter-enhanced representations consistently maintained validation accuracies above 93%, with training accuracy reaching optimal levels by the seventh epoch. Original representations exhibited consistent validation accuracies between 92 and 94%, though with marginally higher variance. The fivefold cross-validation further confirmed these patterns, with diameter-enhanced representations showing the highest consistency across folds, maintaining a mean validation accuracy of 96.8% with a standard deviation of 1.2%.

To provide a comprehensive overview of all classification approaches employed in this study, Table 2 summarizes the performance metrics across all machine learning algorithms, CNN methods, and human expert classifications. This comparison reveals the superior performance of the diameter-enhanced CNN approach and highlights the substantial difference between algorithmic and human expert performance.

**Table 2:** Comparison of classification performance across methods.

Method	Accuracy	Precision	Recall	F1-Score	ROC-AUC	Notes
Machine learning methods						
BART	94.2 ± 2.1	0.91	0.96	0.94	0.97	Bootstrap mean ± SD
C5.0	93.8 ± 2.3	0.90	0.95	0.92	0.96	Bootstrap mean ± SD
Random forest	95.1 ± 1.8	0.93	0.97	0.95	0.98	Bootstrap mean ± SD
XGBoost	94.7 ± 2.0	0.92	0.96	0.94	0.97	Bootstrap mean ± SD
SVM	92.9 ± 2.5	0.89	0.94	0.91	0.95	Bootstrap mean ± SD
EARTH	93.5 ± 2.2	0.90	0.95	0.93	0.96	Bootstrap mean ± SD
CNN methods						
Original representation	93.64	0.89	1.00	0.94	0.95	Single test performance
Diameter-enhanced	98.18	0.96	1.00	0.98	0.98	Single test performance
Soma-focused	94.55	0.92	0.98	0.95	0.96	Single test performance
Human expert performance						
Expert classification	78.3 ± 12.4	0.72	0.81	0.76	0.79	Inter-rater κ = 0.0132

Bootstrap results based on 5000 iterations with 50 cells per group. Human expert performance estimated from inter-rater reliability analysis.

The results demonstrate that machine learning approaches consistently outperformed human expert classification, with the diameter-enhanced CNN method achieving the highest accuracy (98.18%). Notably, human experts showed considerably lower performance (78.3% ± 12.4%) and high variability (κ = 0.0132), underscoring the need for objective, data-driven classification methods.

Cross-validation analysis further confirmed the stability and reliability of our classification approaches. Table 3 presents detailed cross-validation results for both traditional machine learning and CNN methods, demonstrating consistent performance across different validation schemes.

**Table 3:** Cross-validation (CV) performance summary.

Method	2-Fold CV	5-Fold CV	Average standard deviation
CNN methods			
Original	92.8% ± 1.2%	92.4% ± 1.5%	1.35%
Diameter-enhanced	96.2% ± 0.8%	96.8% ± 1.2%	1.00%
Soma-focused	94.1% ± 1.1%	94.3% ± 1.4%	1.25%
Traditional ML			
Random Forest	94.6% ± 1.8%	94.2% ± 2.1%	1.95%
XGBoost	94.1% ± 2.0%	93.8% ± 2.3%	2.15%
SVM	92.4% ± 2.5%	92.1% ± 2.8%	2.65%

Results show mean accuracy ± standard deviation across folds. Lower average standard deviation indicates more stable performance.

The cross-validation results show that diameter-enhanced CNN representations maintained the most stable performance across validation folds (SD = 1.00%), while traditional machine learning methods showed slightly higher variability but remained within acceptable ranges.

The neuron-specific classification analysis revealed particularly noteworthy patterns. Among pyramidal neurons, several specimens consistently presented classification challenges. Notably, pyramidal6aFI was misclassified across all three representation types, while pyramidal1aACC showed misclassification in both original and soma-focused representations as shown in Table 4. The more complex cases, including neurons from series 141–149 and 92–96, maintained high classification accuracy exceeding 92%.

**Table 4:** Misclassified pyramidal neurons across representations: This table identifies pyramidal neurons that were incorrectly classified under three different representation methods: Original, Diameter-Enhanced, and Soma-Focused. Each row represents a specific neuron (identified by its Neuron ID in the leftmost column), and an ‘X’ in any column indicates that the neuron was misclassified using that particular representation method. For example, neuron ‘pyramidal6aFI’ was misclassified in all three representation methods, while ‘pyramidal1aACC’ was only misclassified in the Original and Diameter-Enhanced methods, but correctly classified using the Soma-Focused method.

Neuron ID	Original	Diameter-enhanced	Soma-focused
pyramidal6aFI	X	X	X
pyramidal1aACC	X		X
pyramidal9aFI		X	X
pyramidal14aFI			X
pyramidal15aFI			X
pyramidal7aACC	X		
pyramidal17aFI	X		
D1_GCIA_13-2	X		
D4_GCIA_13-10	X		
D3_GIB3_14-4		X	
D4_GCIA_13-9			X
D4_GCIA_10-3			X

The comprehensive analysis of misclassification patterns revealed that certain pyramidal neurons consistently presented classification challenges across multiple representation types. As shown in Table 2, pyramidal6aFI was the only reconstruction misclassified in all three representations, while others showed representation-specific vulnerabilities. Notably, soma-focused representations demonstrated increased susceptibility to misclassification of pyramidal neurons with VEN-like soma characteristics, particularly in reconstructions pyramidal14aFI and pyramidal15aFI.

Grad-CAM visualization analysis uncovered distinct attention patterns specific to each representation type. In *original* representations, the model exhibited primary attention concentration on the soma region with activation intensities between 0.8 and 1.0, accompanied by strong secondary focus on primary dendrite bifurcations (activation intensities 0.6–0.8). *Diameter-enhanced* representations displayed more distributed activation patterns, with particular emphasis on diameter variations (activation intensities 0.7–0.9) and dendritic branching patterns (activation intensities 0.6–0.8). *Soma-focused* representations demonstrated intense activation localization in the soma region (activation intensities 0.9–1.0), with characteristic gradual attention decay along proximal dendrites.

The systematic masking experiments provided crucial insights into component importance. Soma masking maintained robust performance with 93.64% classification accuracy and an F1-Score of 0.94, despite limited morphological information. Dendrite masking achieved the highest performance among all masking conditions, with 98.18% accuracy and an F1-Score of 0.98. This suggests that while soma features provide sufficient discriminative power for basic classification, dendritic architecture offers additional discriminative features that enhance classification accuracy. The integration of both features in diameter-enhanced representations yielded optimal performance of the Grad-CAM visualization analysis.

Ultimately, combining different approaches to operationalizing neuronal morphology features provided a clear picture, with each method extracting valuable information about the complexity of the features characterizing VENs and pyramidal neurons. Machine learning classifiers, using common morphological measures (Table 1), highlighted dendritic features such as average length, number of stems, and average diameter (Fig. 2). In contrast, Grad-CAM visualization analysis emphasized soma format as the most important feature. Nevertheless, both approaches yielded similar results, demonstrating that both dendritic arborization and soma format exhibit specific alterations in VENs compared to typical pyramidal neurons.

## Discussion

In this study, we analyzed to what extent VENs could be differentiated from other types of pyramidal neurons, utilizing a total of seven classifier models on 761 whole-cell reconstructions from the NeuroMorpho.Org database. Our analysis revealed that, in principle, such a classification should be possible, as indicated by the fact that the classifier models had reasonably high accuracies. Nevertheless, a subset of neuronal morphologies proved to be exceedingly difficult to properly classify, and we have identified the main causes of these misclassifications. Finally, we give recommendations for future research and data collection in order to ensure better consistency and reproducibility of new analyses.

### Supervised machine ensemble learning classification algorithms showed high accuracy in VEN classification

Characterizing morphological features with quantitative measures allows us to obtain information about the importance of each of these measures using both classification algorithms and human expert analysis. The evaluation of the importance of each one of 21 measurements extracted from the NeuroMorpho.Org reconstructions showed low agreement between experts’ answers and machine learning findings. The cognitive complexity of the task^59^, which required the experts to order each measurement, could explain this difference.

From the point of view of the machine learning classifiers, evaluating variable importance was computationally expensive, necessitating a bootstrapping approach to assess all morphological measurements. This process identified a subset of key measurements, primarily associated with dendritic arborization. The latter would be due to the operationalization of reconstructed neuronal morphology in terms of connected cylindrical compartments, when converted into a SWC NeuroMorpho.Org format. While this operationalization is effective for representing structures that typically exhibit a cylindrical shape, such as dendritic and axonal arborization, its applicability is limited when dealing with structures that deviate from this form, including the neural soma. This limitation could account for the lower importance assigned to soma-related measurements compared to those encompassing all dendritic extensions.

Regarding neuronal reconstruction classification, the machine learning classifiers exhibited high accuracy and consistency across different algorithms. Although a group of neuronal reconstructions was misclassified by all classifiers, this suggests that intermediate or subtle morphological features, not directly captured by the standard NeuroMorpho.org measurements, may be necessary to fully differentiate VENs from pyramidal neurons. It is also possible that at least some of these neurons were mislabeled.

### Grad-CAM visualization analysis revealed the relevance of both somatic and dendritic morphology in distinguishing VENs from other pyramidal neurons

The classification performance across different neuronal representations reveals significant insights into both methodological and biological aspects of VEN identification. The superior performance achieved with diameter-enhanced representations (98.18% accuracy) strongly suggests that dendritic diameter variations serve as crucial diagnostic features for VEN identification, aligning with recent morphological studies^28,60^. Notably, the model’s maintained high performance under soma-masked conditions challenges traditional classification approaches that heavily emphasize soma morphology, suggesting that dendritic architecture alone carries substantial discriminative power.

The consistent misclassification patterns observed in specific pyramidal neurons may suggest the existence of a morphological continuum between VENs and certain pyramidal neurons, rather than the existence of strictly separate categories, potentially reflecting evolutionary or developmental relationships that warrant further investigation^57,58^. This observation has important implications for our understanding of neuronal type evolution and development, potentially supporting theories about the specialized adaptation of certain cortical neurons^26,61^. The higher misclassification rates in soma-focused representations of certain pyramidal neurons (e.g., pyramidal14aFI and pyramidal15aFI) suggest that soma morphology alone may be insufficient for definitive classification in borderline cases.

Grad-CAM visualization analysis provides novel insights into the model’s decision-making process, revealing biologically relevant feature recognition patterns. The strong activation in soma regions aligns with traditional morphological criteria and is consistent with recent research showing the importance of soma morphology in delineating cortical regions and layers^62,63^. However, the significance of dendritic branching patterns should not be neglected, and our analysis revealed the importance of these aspects of neuronal morphology. These findings complement recent studies on neuronal morphology classification^64^ and provide quantitative support for expanding classification criteria beyond soma-centric approaches.

The technical success of the VGG-based approach with transfer learning demonstrates the viability of deep learning for specialized neuronal classification tasks. This methodology’s effectiveness, particularly in handling various neuronal representations, suggests potential applications in broader neuromorphological studies. The model’s ability to maintain high performance across different visualization techniques (original, diameter-enhanced, and soma-focused) indicates robust feature extraction capabilities that could be valuable for analyzing other specialized neuron types.

The differences between supervised machine learning algorithms and Convolutional neural network classifications show that neither approach can identify all morphological features. Therefore, further research to develop a better three-dimensional representation for non-cylindrical shapes, like the soma, could be essential for evaluating its significance. Similarly, creating better measurements to identify specific characteristics of non-cylindrical shapes will also be beneficial.

These findings present several important considerations for future research directions. The relationship between 2D representations and actual 3D neuronal structure remains a crucial area for investigation, as does the impact of various visualization techniques on classification accuracy. In contrast to Grad-CAM visualization, quantifying morphological features provides an alternative approach to studying neuronal morphology, highlighting dendritic characteristics that the visualization method missed. Therefore, combining different machine learning approaches may capture the full complexity of neuronal morphology.

Nevertheless, the balance between automated classification and expert validation requires careful consideration, particularly in cases where morphological features show intermediate characteristics. Future studies might benefit from investigating misclassified cases as potential sources of insight into novel morphological variants or transitional forms between neuron types.

The implications of these findings extend beyond technical achievements in neuronal classification. They suggest a need for refined understanding of neuronal type boundaries and highlight the potential value of quantitative morphological analysis in neuroscience. The model’s ability to identify subtle morphological patterns could aid in understanding the relationship between neuronal form and function, particularly in specialized cortical neurons such as VENs. This understanding becomes especially relevant in the context of neurological conditions where VEN abnormalities have been implicated^24,65^.

Understanding these various aspects provides crucial context for interpreting our results and suggests multiple avenues for future investigation. The integration of deep learning approaches with traditional neuromorphological expertise may offer new perspectives on neuronal classification and development, while also providing practical tools for research and clinical applications.

Our findings demonstrate that VENs can, in principle, be distinguished from other types of pyramidal neurons using machine learning, which could greatly aid the current classification methods that are predominantly expert-driven. Importantly, our results implicate that dendritic architecture can offer discriminative power comparable to soma morphology, challenging the soma-centric view prevalent in much of the literature. This implies that VEN classification protocols should be broadened to incorporate dendritic features as standard criteria, at least in borderline cases and in cases where VENs are being described in new species, cortical regions, or cortical layers. Furthermore, we identified consistent sources of misclassification, many of which relate to methodological artifacts or inconsistent original labeling. This underscores the necessity of more rigorous data quality control before uploading data to neuromorphological repositories. Together, these insights establish a methodological framework that can improve the accuracy, reproducibility, and comparability of VEN research across different research teams.

### The rationale behind algorithm-based VEN classification and potential ramifications

Existing research suggest that VENs are metabolically costly neurons, thought to promote the rapid relay of information across large distances^66,67^. Combined with the observation that their numbers are elevated in humans relative to other non-human primates^68^, these properties suggest that VENs might have been favored during hominid evolution, potentially playing a prominent role in human cognition^17,27^. This view is reinforced by the localization of the majority of VENs in humans to the insular and cingulate cortices^17^—both of which belong to a small set of cortical regions consistently (co-)activated across a wide range of neurocognitive processes^69,70^.

A substantial body of evidence further indicates that both the insular and cingulate cortices comprise multiple functionally distinct subregions. The insula, for instance, can be divided into dorsal anterior and ventral anterior subdivisions (dAI, vAI) as well as a posterior subdivision (pIns;^71^), while the cingulate cortex can be differentiated into anterior cingulate (ACC), midcingulate (MCC), posterior cingulate (PCC) and retrosplenial cortices^72^. Drawing on data from the Human Connectome Project (HCP), Cai and Menon^73^ recently demonstrated that the dAI is most closely associated with working memory, whereas the vAI appears more strongly linked to social and emotional aspects of cognition. The pIns, by contrast, is primarily involved in sensorimotor processing. Similarly, the subgenual anterior cingulate cortex (sgACC) has been linked to affective and autonomic functions, whereas the dorsal ACC/midcingulate cortex (MCC) is a well-established hub of cognitive control and working memory^72,74,75^.

Since VENs have been described as most abundant in the vAI and ACC^17,76^, their functional roles have frequently been equated with those of these subregions^1^. However, the identification of VENs has thus far relied on the subjective judgement of individual researchers rather than objective classification criteria, raising the risk of biased or arbitrary functional assignments. The potential consequences of this limitation are illustrated by^77^, who conducted a postmortem investigation of neuronal factors underlying the exceptional mnestic abilities of so-called “SuperAgers”—older adults whose cognitive performance matches that of individuals decades younger. They found that SuperAgers differed from both cognitively average elderly controls and individuals with mild amnestic cognitive impairment (aMCI) only in VEN density within the ACC, despite all groups showing the antero–caudal VEN gradient previously reported in the literature^61^. These findings may suggest that the relationship between VENs and cognition is more complex and diverse than previously assumed. However, the core issue is the uncertainty over whether a given spindle-shaped neuron is a VEN or a morphologically similar common MPN: the two may overlap morphologically, but nevertheless fulfil unrelated functional purposes.

If the identity of VENs were to be established conclusively, it would become possible to examine precisely how they contribute to specific behaviors and cognitive processes in both neurological disease and health, potentially informing the development of targeted interventions involving VENs and their circuits. Achieving this goal, however, depends on establishing clear, objective procedures as put forward in the present analysis for determining when “a VEN is a VEN.”

While our study focused on binary classification between VENs and pyramidal neurons to address the specific debate about VEN identification criteria, the methodology could be extended to multi-class classification scenarios. Such extension would require modifying the final CNN layer for n-class output and sufficient training samples for each neuron type. Given our high accuracy in binary classification and the identified importance of both dendritic and somatic features, we anticipate that multi-class performance would depend primarily on the morphological distinctiveness between neuron types and data availability. Future studies could leverage our dual approach of morphometric analysis and deep learning to develop comprehensive neuronal classification systems encompassing multiple cortical neuron types.

### Recommendations for future research

In order to make future research both more reproducible and comparable, we propose several guidelines for improving repositories for neuronal reconstructions.

Firstly, we recommend conducting basic checks on reconstruction quality before conversion to a common file format (e.g. .SWC) to avoid human errors derived from the reconstruction process itself. Such checks should include at least the following: (1) checking whether the soma is completely reconstructed in 3D and not cut on the slide edge; (2) checking whether the contour of the soma is closed in all focal planes; (3) checking whether all processes in the reconstruction are attached to the soma; (4) checking whether all processes in the reconstruction are in correct alignment with the soma and with each other (it is particularly important to check for Z drift); and (5) checking whether all processes have appropriate endings marked.

Secondly, we recommend conducting basic checks on reconstruction quality after conversion to a common file format to ensure no errors are introduced during the conversion process; for example, we strongly recommend using software like the web-based neuron morphology viewer (^78^ https://neuroinformatics.nl/HBP/morphology-viewer/) or NeuroEditor^51^ to verify the accuracy of the conversion from the original reconstruction format (e.g., Neurolucida) to SWC.

Thirdly, we recommend depositing relevant morphometric data alongside the reconstruction files themselves. This would enable researchers to evaluate whether the morphometric data derived from the reconstruction files is reliable, i.e. whether distortion occurred or whether reconstruction errors are present. We also recommend collecting additional metadata on the specimens from which the reconstructions are derived (e.g. postmortem delay, age at death, sex, relevant pathology, issues/features related to impregnation and/or reconstruction process, etc.) to be able to assess how these factors potentially affect the analyses of the reconstructions.

Finally, we recommend utilizing more objective and reliable methods of morphological classification in future neuroanatomical studies. Our findings support the notion that combining machine learning models with human expertise can provide a more consistent result in morphological interpretation than relying solely on human expertise which suffers from high interrater variability. However, human expertise is still necessary for final evaluations, since classifier models currently cannot adequately account for various methodological errors that hinder correct assessment.

### Limitations of the study and prospects for future research

It has been shown that various aspects of cell morphology serve as viable tools for neuronal classification^79^; therefore, the present research’s focus rested solely on the role of various morphological properties. Nevertheless, multiple lines of evidence suggest the need to complement and combine our framework with other instructive approaches such as electrophysiology and transcriptomics to facilitate a comprehensive characterization of VENs^13,21^. For instance, in employing single-nucleus RNA sequencing, Hodge et al.^80^ identified a cluster of human neurons in layer Vb, most likely encompassing VENs, fork cells (sporadically co-occurring with VENs), as well as a subpopulation of pyramidal neurons. A comparative analysis based on the well-established transcriptomic profiling of the mouse brain led the authors to propose that said cluster may consist primarily of subcerebrally projecting excitatory neurons (L5 extra-telencephalic [ET] neurons;^80^). Thus, instead of constituting a separate neuron class, VENs may represent a variant of a cell type that was conserved during evolution. At the same time, VENs may have undergone considerable species-specific adaptations. Recently, Yuan et al.^81^ generated profiles of genetic regulation and expression in both the human ACC and that of the macaque. Besides documenting that VENs were likely part of an ET cluster evincing considerable cross-species communalities, their results point to the existence of marker genes specific to human VENs, associated with developmental morphogenesis^81^. Evidently, future extensions of our approach, including the abovementioned features, may aid not only in the classification of human VENs but also in describing how these unique cells differ between species in terms of structure and, ultimately, function.

Despite its considerable merit, our study has several limitations. Firstly, the neuronal metrics that form the basis of our investigation were derived predominantly from spindle-shaped neurons (putative VENs) in the ACC. This may be surprising, given that VENs in humans appear to be more numerous in the fronto-insular cortex (FI) than in the ACC^61^. This discrepancy could be, at least partly, caused by difficulty in obtaining adequate tissue samples of the FI. Namely, the frontoinsular area (F*J*), as described by von Economo and Koskinas^15^, lies at the transition between the frontal and insular lobes and is contained within the transverse insular gyrus. Acquiring histological sections perpendicular to the orientation of the gyrus, which is necessary for proper morphological analysis, may be difficult, because the gyrus is often cut obliquely on standard serial frontal sections. Nevertheless, previous estimates of morphological parameters of VENs in both the ACC and FI have yielded widely comparable results^82^, and we do not consider this limitation to impugn the conclusions presented here. However, we recommend that any replication and/or extension of our analyses include VENs from the FI to provide a fully representative account of human VEN morphology. A further limitation concerns demographic features pertaining to the brain tissue donors that could potentially affect the generalizability of our results. The bulk of the VEN reconstructions used in our analyses were based on neurons obtained from younger males (age range: 18 to 59), highlighting the need to extend our methodology to samples consisting of senescent and/or female subjects. Moreover, as various lines of evidence point to the co-occurrence of abnormalities related to VENs (including, but not limited to their morphology) with various neuropsychiatric disorders, future studies should attempt to replicate our results using quality data from various subsamples differing with respect to their mental status.

### Conclusion

As many questions regarding the cortical distribution of VENs remain unanswered, we contend that the future application of the procedures put forth in this study may shed light on this prominent issue. For instance, reexamining the identity of spindle-shaped neurons previously labelled as VENs in regions where they had not originally been described by von Economo, such as the DLPFC and precuneus, may help unambiguously delineate the presence of VENs in these areas. The resultant refined localization of VENs, in turn, may aid in disentangling the potential functional implications of VENs. Moreover, we have demonstrated that the prevailing reliance on soma features when identifying VENs may fail to capture important discriminative properties inherent in the structural organization of dendritic branches. Especially when handling neuromorphological fringe cases, this information may aid in improving classification accuracy.

In conclusion, this study not only demonstrates that machine learning models can achieve high accuracy in differentiating VENs from pyramidal neurons, but also highlights specific morphological parameters and methodological considerations that are critical for reliable classification. Crucially, we demonstrate that machine learning models have a higher reliability than human expert raters in determining which morphological features are the most relevant for VEN classification. This is consistent with other research comparing machine learning models and expert performance in similar settings (e.g. cortical layer delineation)^62^, implicating that it would be prudent to utilize machine learning in future research, at least as a supplement to human experts. By highlighting specific dendritic and somatic parameters as key discriminators, and by identifying common sources of classification error, we provide a replicable, data-driven framework for VEN identification. This framework can be applied to re-evaluate existing datasets, inform future histological investigations, and ultimately advance our understanding of VEN distribution and function in both health and disease.

## Supplementary Information

Below is the link to the electronic supplementary material.

Supplementary Material 1

Supplementary Material 2

Supplementary Material 3

Supplementary Material 4

## Data Availability

All data and R codes are available at the following repository: https://github.com/julian-tejada/morphologycalAnalysisVENs.
