# Supplementary material for: VENturing into machine learning for the morphological analysis of von Economo neurons
Source: Sci Rep. 2025 Dec 20;15:45033. doi: 10.1038/s41598-025-30470-y (PMC12749959; doi:10.1038/s41598-025-30470-y)
Supplement: Supplementary file 1 — Supplementary Material 1 [file 41598_2025_30470_MOESM1_ESM.docx]

**Supplementary material**

**Supplementary tables**

**Supplementary Table 1**. Operationalization of the morphometric parameters analyzed in this study according to the L-measure help documentation.

| **Parameter** | **Description** | **Image** |
| --- | --- | --- |
| Average Rall’s ratio | The best value that fits the equation Bif_Dia)^Rall=(Daughter1_dia^Rall+Daughter2_dia^Rall), bearing in mind that a daughter is a branch that has sprouted after a bifurcation. | 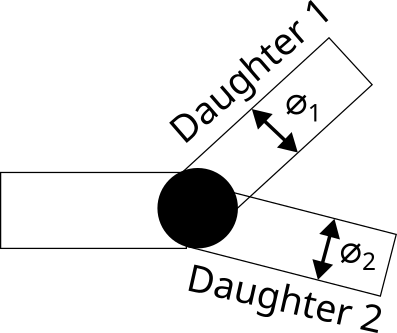 |
| Average bifurcation angle local | The angle, in degrees, between the first two compartments after a bifurcation. | **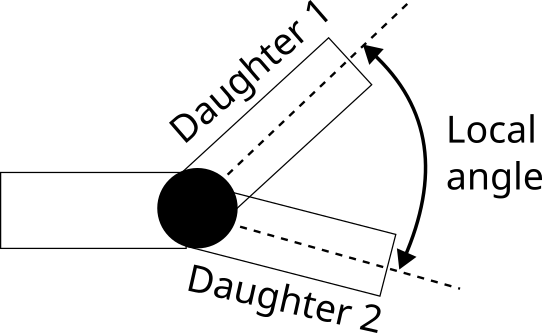** |
| Average bifurcation angle remote | The angle, in degrees, between the end of two branches that share the same bifurcation. | 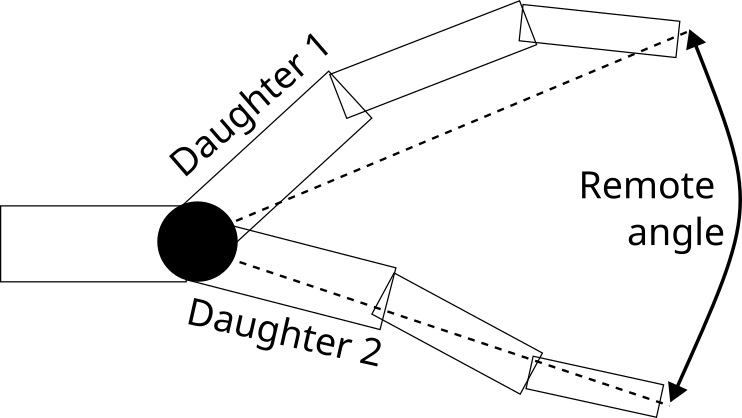 |
| Average contraction | The ratio between the Euclidean distance between two bifurcations and their path length (sum of compartment lengths). | 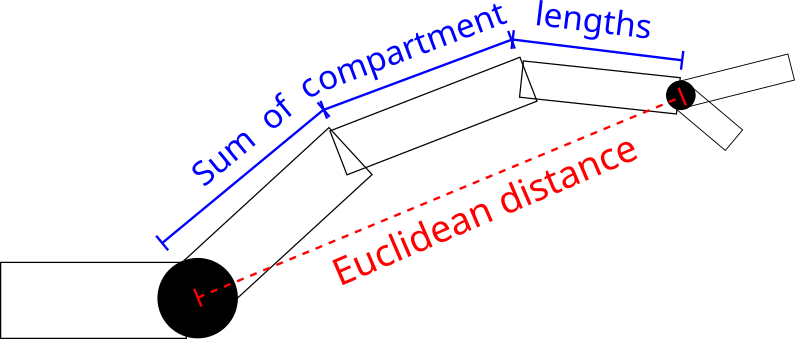 |
| Average diameter | The average diameter considering all compartments (soma, dendrites, axons, etc) of the neuron, in microns. | 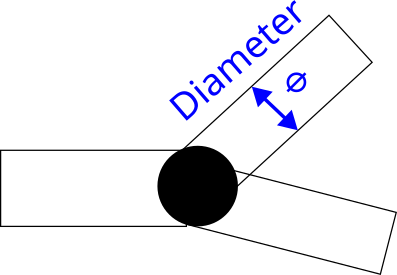 |
| Average fragmentation | The average number of compartments composed of a branch between two bifurcations. | 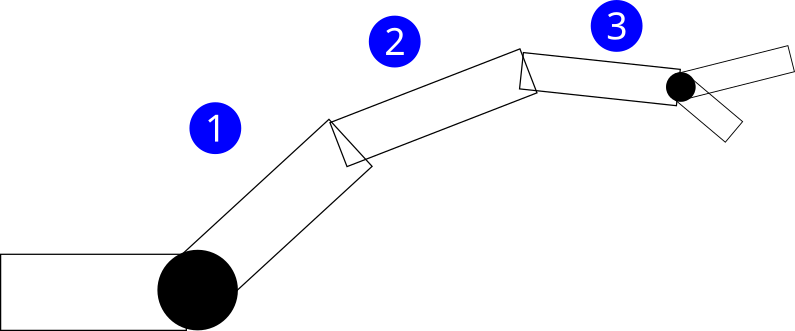 |
| Average length | The average length between the onset and end point of each compartment, in microns. | 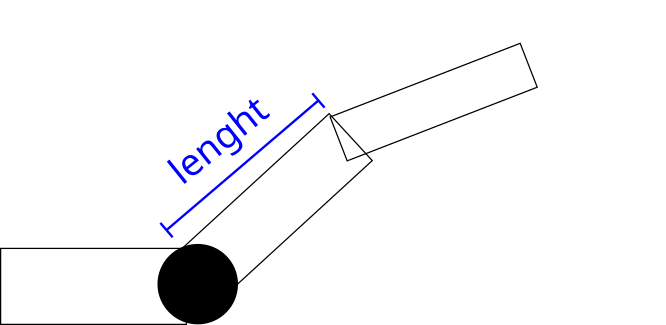 |
| Branch order | The total sum of the centrifugal branch order of all the branches with respect to soma, where soma has order=0, first bifurcation has order=1, second has order=2 and so on. |  |
| Max Euclidean distance | The maximum straight line distance from the soma to the end point of each compartment, in microns. | 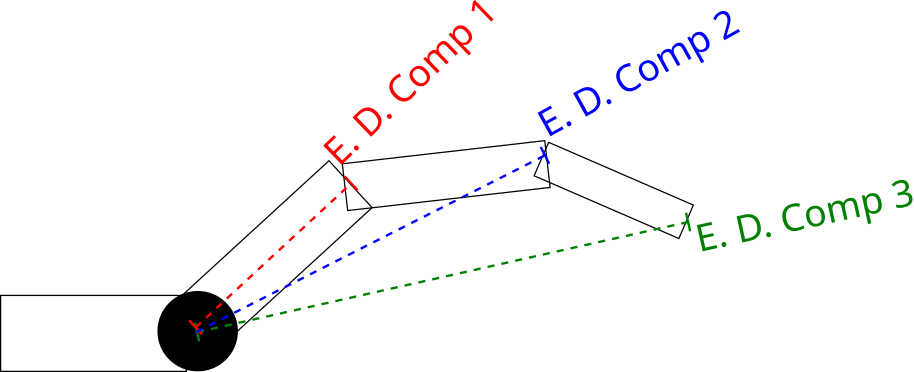 |
| Average Euclidean distance | The average straight line distance from the soma to the end point of each compartment, in microns. | 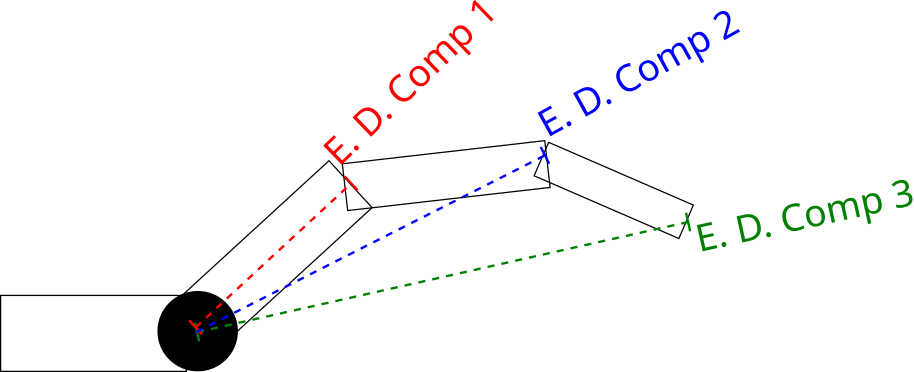 |
| Max branch order | The maximum branch order. |  |
| Max path distance | The maximum of all path lengths between two bifurcations, in microns. | 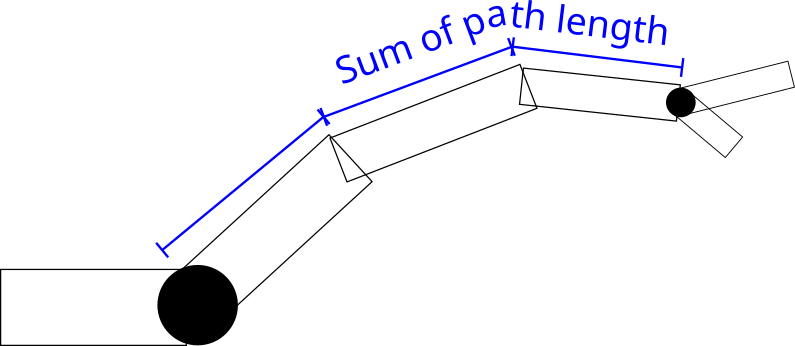 |
| Number of bifurcations | The total number of bifurcations. |  |
| Number of branches | The total number of branches. |  |
| Number of stems | The total number of compartments arising from the soma. |  |
| Overall depth | The truncated difference of the minimum and maximum z-values, after eliminating the outer points, in microns. | 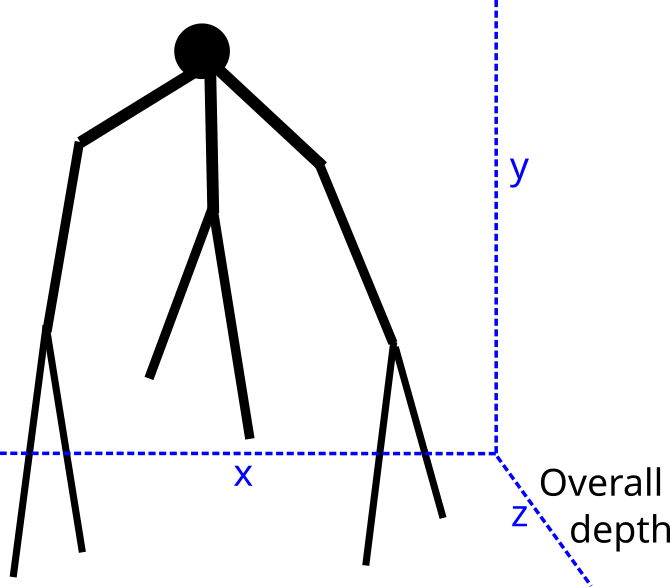 |
| Overall height | The truncated difference of the minimum and maximum y-values, after eliminating the outer points, in microns. | 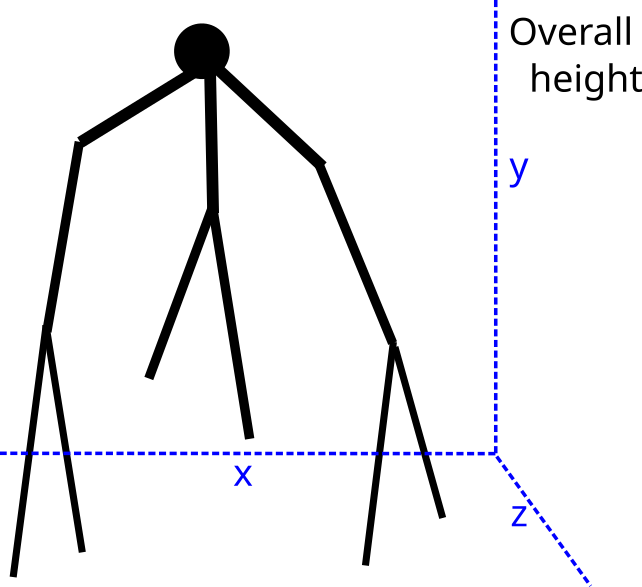 |
| Overall width | The truncated difference of the minimum and maximum x-values, after eliminating the outer points, in microns. | 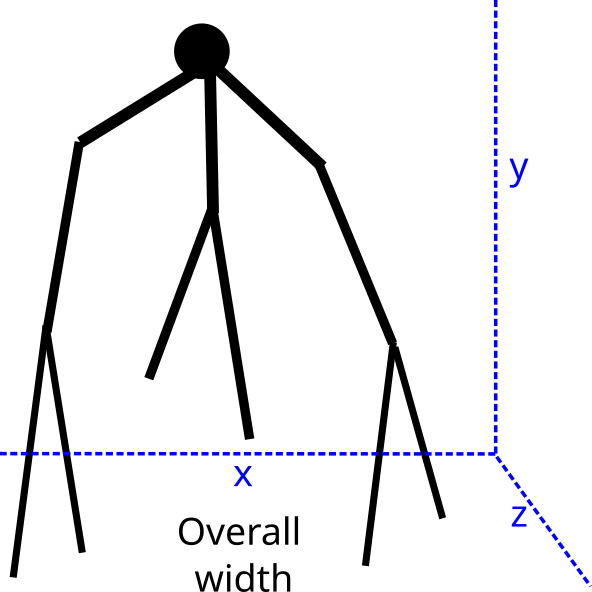 |
| Partition asymmetry | The average of the result of the equation abs(n1-n2)/(n1+n2-2), where n1 is the number of tips (terminal compartments with no further branching) on the left and n2 on the right in a given bifurcation. | 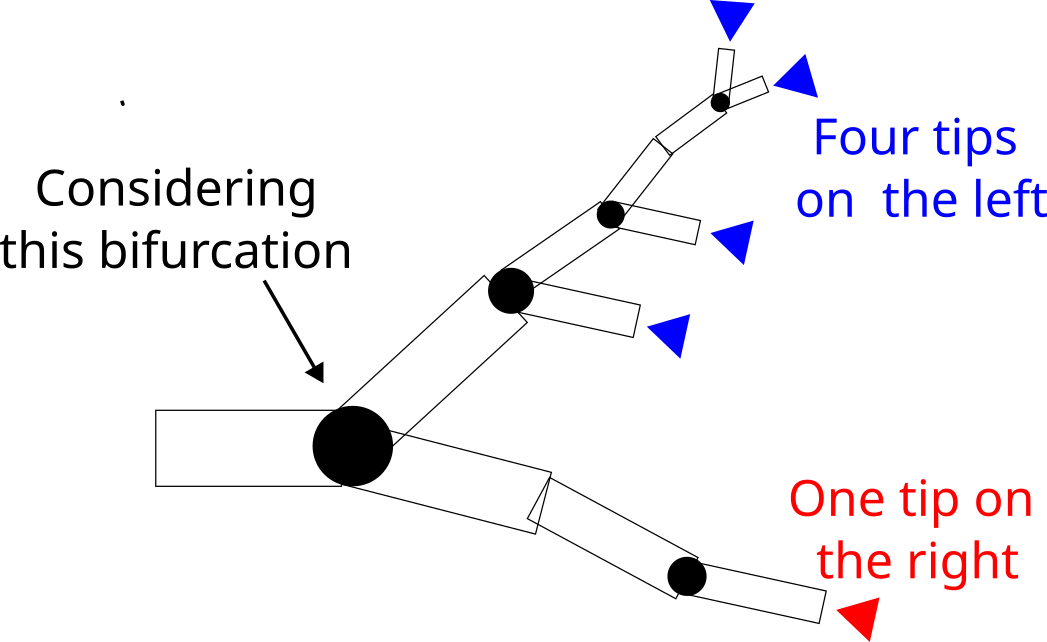 |
| Soma surface | The surface of the compartments that form the soma, in microns. |  |
| Soma height | The truncated difference of the minimum and maximum y-values of the soma compartment, in microns. |  |
| Soma width | The truncated difference of the minimum and maximum x-values of the soma compartment, in microns. |  |
| Total surface | The sum of the surface of all compartments that form the neuron, in microns. |  |
| Total volume | The sum of the volume of all compartments that form the neuron, in cubic microns. |  |
| Overall height-to-width ratio | Overall height/overall width. |  |
| Soma height-to-width ratio | Soma height/soma width. |  |

##

**Supplementary Table 2.** List of misclassified reconstructions during the information-driven variable selection. The cell name corresponds with the reconstruction name registered by the researchers in neuromorpho.org. The cell type corresponds to the type of cell registered in NeuroMorpho.org.

[Supplementary Table 2.xlsx](https://docs.google.com/spreadsheets/d/1tUoj-WJtvkh5ZTA4tehrkwABfmN-lA3X/edit?usp=drive_link&ouid=109727986698846215648&rtpof=true&sd=true)

**Supplementary Table 3.** Variance analysis: machine learning vs human experts

| **Feature Ranking Position** | **ML Algorithms Variance** | **Human Experts Variance** | **Ratio (Human/ML)** |
| --- | --- | --- | --- |
| 1^st^ | 0.12 | 2.84 | 23.67 |
| 2^nd^ | 0.18 | 3.21 | 17.83 |
| 3^rd^ | 0.23 | 2.97 | 12.91 |
| 4^th^ | 0.31 | 3.45 | 11.13 |
| 5^th^ | 0.28 | 3.12 | 11.14 |
| Overall Variance | 0.22 ± 0.07 | 3.12 ± 0.23 | 14.18 |

*Variance calculated across ranking positions for morphological feature importance. Lower variance indicates higher consistency. Ratio > 1 indicates human experts show greater variability than ML algorithms.*

**Supplementary Text**

*Gradient-weighted Class Activation Mapping (Grad-CAM)*

To provide interpretability and insight into the network's decision-making process, we implemented Gradient-weighted Class Activation Mapping (Grad-CAM) (Selvaraju et al., 2017, 2020). Grad-CAM generates class-discriminative localization maps highlighting regions that strongly influence the model's classifications. The technique operates by:

1. Computing the gradient of the target class score with respect to feature maps of the final convolutional layer
2. Global-average-pooling these gradients to obtain neuron importance weights
3. Performing a weighted combination of forward activation maps
4. Applying ReLU to eliminate negative influences

Mathematically, for a given class c, the class-discriminative localization map ${L^{c}}_{Grad-CAM}$ is computed as:

αᶜₖ = (1/Z) ∑ᵢ ∑ⱼ (∂yᶜ/∂Aᵏᵢⱼ)

$${L^{c}}_{Grad-CAM}= ReLU(\Sigma_{k} {\alpha^{c}}_{k} A^{k})$$

where ${\alpha^{c}}_{k}$represents the importance weights, $A^{k}$represents the feature maps, and $y^{c}$ is the score for class c before the softmax (Selvaraju et al., 2020).

We focused our Grad-CAM analysis on the final convolutional layer (layer 40) of the VGG16-BN network, as this layer captures high-level features while maintaining sufficient spatial resolution for meaningful localization (Zhou et al., 2016). The resulting heatmaps were upsampled to match the input image dimensions using bilinear interpolation.

### *Component-Specific Analysis through Neuronal Masking*

The morphological complexity of Von Economo Neurons presents a unique challenge in understanding which structural components contribute most significantly to their classification. To address this challenge, we developed a systematic masking protocol that enables the isolation and analysis of specific neuronal components. This approach was designed to disentangle the relative contributions of soma architecture and dendritic arborization patterns in the classification process.

The masking protocol implemented three distinct visualization paradigms: complete neuronal visualization, soma-specific analysis, and dendrite-focused examination. For the soma-specific analysis, we employed a circular masking function centered on the cell body, defined mathematically as:

M(x,y) = { 1, if (x-x_c)² + (y-y_c)² ≤ r² 0, otherwise }

where (x_c, y_c) represents the soma centroid coordinates, and r is dynamically calculated as one-fourth of the minimum dimension of the input image. This ratio was empirically determined through preliminary experiments to optimally encompass the soma while minimizing inclusion of proximal dendritic segments. The mask was applied as a binary operator, effectively isolating the soma region while nullifying all other neuronal components.

For the dendrite-focused analysis, we employed the inverse of the soma mask, effectively removing the cell body while preserving the complete dendritic arbor. This complementary approach allows for the assessment of dendritic morphology's contribution to neuronal classification independent of soma characteristics. The mathematical representation of the dendrite mask can be expressed as:

D(x,y) = 1 - M(x,y)

The objective of this dual-masking approach was threefold. First, it enables the quantitative assessment of which neuronal components carry the most discriminative information for VEN classification. Second, it provides insight into whether the network's classification decisions align with established morphological criteria used by expert neuroanatomists. Third, it helps validate whether the model is learning genuine morphological features rather than artifacts or spurious correlations in the data.

This masking strategy is particularly relevant given the ongoing debate in the field regarding the definitive characteristics of VENs (Cauda et al., 2014; Petanjek et al., 2023). By systematically isolating and analyzing different neuronal components, we can provide quantitative evidence regarding which morphological features are most diagnostic for VEN classification. This approach may help establish more objective criteria for VEN identification, addressing a crucial need in the field.

### *Grad-CAM Implementation and Justification*

The selection of Gradient-weighted Class Activation Mapping (Grad-CAM) as our primary visualization technique was motivated by several key considerations. First, unlike other interpretation methods such as simple CAM or Guided Backpropagation, Grad-CAM provides class-discriminative localization without requiring architectural modifications or retraining (Selvaraju et al., 2020). This was particularly important for our transfer learning approach, as it allowed us to maintain the integrity of the pre-trained VGG16 architecture while still gaining insight into its decision-making process.

Second, Grad-CAM's ability to generate coarse localization maps aligns well with the spatial scale of neuronal features we aimed to analyze. The technique produces activation maps that highlight regions of interest while maintaining sufficient resolution to distinguish between major neuronal components. This characteristic is especially valuable when analyzing the relative contributions of soma versus dendritic features in the classification process.

We implemented Grad-CAM by computing the gradient of the target class score y^c with respect to feature maps A^k of the final convolutional layer:

α^c_k = (1/Z) Σ_i Σ_j (∂y^c/∂A^k_ij)

where α^c_k represents the importance weights for each feature map k. The class-discriminative localization map was then generated through a weighted combination of forward activation maps:

L^c_Grad-CAM = ReLU(Σ_k α^c_k A^k)

The ReLU function was applied to eliminate negative influences, focusing only on features that positively contribute to the class of interest. This approach allows us to visualize specifically which regions of the neuron most strongly influence the network's classification decision.

When combined with our masking protocol, Grad-CAM provides a powerful tool for understanding not only which regions of the neuron are important for classification, but also how the network's attention patterns shift when certain components are isolated or removed. This dual analysis approach enables us to:

1. Validate whether the network's attention aligns with known morphological criteria for VEN identification
2. Identify potentially novel discriminative features that may not be apparent through traditional manual inspection
3. Quantify the relative importance of different neuronal components in the classification process
4. Assess the robustness of the classification when certain morphological features are obscured

The integration of Grad-CAM visualization with systematic masking provides a comprehensive framework for understanding both the what and the why of the network's classification decisions. This approach not only validates the model's performance but also contributes to the broader scientific understanding of VEN morphology and classification criteria.

**References**

Selvaraju, R. R., Cogswell, M., Das, A., Vedantam, R., Parikh, D., & Batra, D. (2017). Grad-CAM: Visual Explanations from Deep Networks via Gradient-Based Localization. 2017 IEEE International Conference on Computer Vision (ICCV), 618–626. https://doi.org/10.1109/ICCV.2017.74

Zhou, B., Khosla, A., Lapedriza, A., Oliva, A., & Torralba, A. (2016). Learning Deep Features for Discriminative Localization. 2016 IEEE Conference on Computer Vision and Pattern Recognition (CVPR), 2921–2929. https://doi.org/10.1109/CVPR.2016.319

**Supplementary figure descriptions**

**Supplementary figure 1** – Examples of misclassified reconstructions. (**A**) A typical VEN with a high level of soma distortion present in the NeuroMorpho.org .SWC file. Note the circled globular shape of the soma, which is not present in the original publication. (**B**) An example of Z-drift shifting the plane of the apical dendrite away from the plane of the soma. The arrow marks the unusual bend in the proximal portions of the apical dendrite. (C) A cell with likely incomplete impregnation of dendritic arbor. Note the lack of side dendrites arising from the some as well as the poor branching complexity of both the apical and basal dendritic trees.

**Supplementary figure 2** - Examples of misclassified reconstructions. (**A**) A pyramidal neuron with a cut or incompletely reconstructed apical dendrite. The arrow marks the abrupt end of the apical dendrite. (**B**) A cell with unusual somatic and dendritic morphology, misclassified as a VEN. The pentagon left to the cell highlights the polygonal shape of this cell’s soma, which corresponds neither to typical pyramidal neurons, nor to VENs. (**C**) Bipolar pyramidal neuron probably mislabeled as a VEN in the NeuroMorpho.org database. The pyramidal soma is circled, and the arrow denotes the third major dendrite arising from the third corner of the soma.
